# The role of the soft palate dose regarding normal tissue toxicities in older adults with head and neck cancer undergoing definitive radiotherapy

**DOI:** 10.1186/s13014-024-02426-5

**Published:** 2024-04-30

**Authors:** Helena C. Bitz, Ilias Sachpazidis, Jiadai Zou, Daniel Schnell, Dimos Baltas, Anca-Ligia Grosu, Nils H. Nicolay, Alexander Rühle

**Affiliations:** 1 Department of Radiation Oncology, Medical Center, Faculty of Medicine, University of Freiburg, Robert-Koch-Str. 3, 79106 Freiburg, Germany; 2 German Cancer Consortium (DKTK) Partner Site Freiburg, German Cancer Research Center (DKFZ), Heidelberg, Germany; 3 Division of Medical Physics, Department of Radiation Oncology, Medical Center, Faculty of Medicine, University of Freiburg, Freiburg, Germany; 4 Department of Radiation Oncology, University of Leipzig, Leipzig, Germany; 5 Comprehensive Cancer Center Central Germany, Partner Site Leipzig, Leipzig, Germany

**Keywords:** Normal tissue complication probability, NTCP, Chemoradiation, HNSCC, Xerostomia, Dysgeusia, Dysphagia, Geriatric, Elderly

## Abstract

**Purpose:**

The number of older adults with head and neck squamous cell carcinoma (HNSCC) is continuously increasing. Older HNSCC patients may be more vulnerable to radiotherapy-related toxicities, so that extrapolation of available normal tissue complication probability (NTCP) models to this population may not be appropriate. Hence, we aimed to investigate the correlation between organ at risk (OAR) doses and chronic toxicities in older patients with HNSCC undergoing definitive radiotherapy.

**Methods:**

Patients treated with definitive radiotherapy, either alone or with concomitant systemic treatment, between 2009 and 2019 in a large tertiary cancer center were eligible for this analysis. OARs were contoured based on international consensus guidelines, and EQD2 doses using α/ß values of 3 Gy for late effects were calculated based on the radiation treatment plans. Treatment-related toxicities were graded according to Common Terminology Criteria for Adverse Events version 5.0. Logistic regression analyses were carried out, and NTCP models were developed and internally validated using the bootstrapping method.

**Results:**

A total of 180 patients with a median age of 73 years fulfilled the inclusion criteria and were analyzed. Seventy-three patients developed chronic moderate xerostomia (grade 2), 34 moderate dysgeusia (grade 2), and 59 moderate-to-severe (grade 2–3) dysphagia after definitive radiotherapy. The soft palate dose was significantly associated with all analyzed toxicities (xerostomia: OR = 1.028, dysgeusia: OR = 1.022, dysphagia: OR = 1.027) in the multivariable regression. The superior pharyngeal constrictor muscle was also significantly related to chronic dysphagia (OR = 1.030). Consecutively developed and internally validated NTCP models were predictive for the analyzed toxicities (optimism-corrected AUCs after bootstrapping: AUC_xerostomia_=0.64, AUC_dysgeusia_=0.60, AUC_dysphagia_=0.64).

**Conclusions:**

Our data suggest that the dose to the soft palate is associated with chronic moderate xerostomia, moderate dysgeusia and moderate-to-severe dysphagia in older HNSCC patients undergoing definitive radiotherapy. If validated in external studies, efforts should be undertaken to reduce the soft palate dose in these patients.

**Supplementary Information:**

The online version contains supplementary material available at 10.1186/s13014-024-02426-5.

## Introduction

Head and neck squamous cell carcinoma (HNSCC) is the sixth most common malignancy worldwide, causing significant morbidity and mortality [1]. Older patients with HNSCC face unique treatment challenges due to underrepresentation in clinical trials and therefore limited evidence [2]. There is an urgent need to increase scientific evidence for optimal management of these patients, as the number of older HNSCC patients will further increase in the next decades [3, 4].

Surgery and radiotherapy are the main treatment modalities for patients with localized HNSCC [5]. Although a matter of debate in the older HNSCC population [6–8], concomitant chemotherapy is commonly applied simultaneously to definitive radiotherapy for locoregionally advanced HNSCCs. Radiotherapy can result in considerable both acute and chronic toxicities in HNSCC patients, severely impacting patients’ quality of life (QoL) [9, 10], conformal treatment techniques such as intensity-modulated radiotherapy (IMRT) have been shown to reduce treatment-related toxicities, as relevant organs at risks (OARs) such as the parotid glands or the pharyngeal constrictor muscles (PCMs) can be spared [11, 12]. Proton IMRT may further reduce the risk of treatment-induced normal tissue injuries in HNSCC patients and is currently investigated in clinical trials [13, 14]. However, xerostomia, dysgeusia and dysphagia are still among the most prevalent and QoL-affecting toxicities in long-term HNSCC survivors after radiotherapy [15–17].

Older HNSCC patients may be more susceptible to chronic treatment-related adverse events due to lower functional reserves [18]. Besides differences in the vulnerability to treatment-induced toxicities. physiological aging processes as well as polypharmacy and comorbidities may result in higher rates of treatment-related normal tissue toxicities [19–22]. For instance, Sommers and colleagues reported in a conference abstract that the prevalence for dysphagia grade ≥ 2 and severe xerostomia was higher than expected in older HNSCC patients, therefore requiring adjustments of the comprehensive individual toxicity risk (CITOR) model [23].

Normal tissue complication probability (NTCP) models may aid radiation oncologists and radiation physicists in the radiation treatment planning process [24–27]. As available NTCP models were developed and validated for the general HNSCC population, we aimed to examine the association between OAR doses and the common chronic toxicities xerostomia, dysgeusia and dysphagia specifically in the older HNSCC population using modified NTCP models.

## Materials and methods

### Patients and treatment

**Fig. 1:**
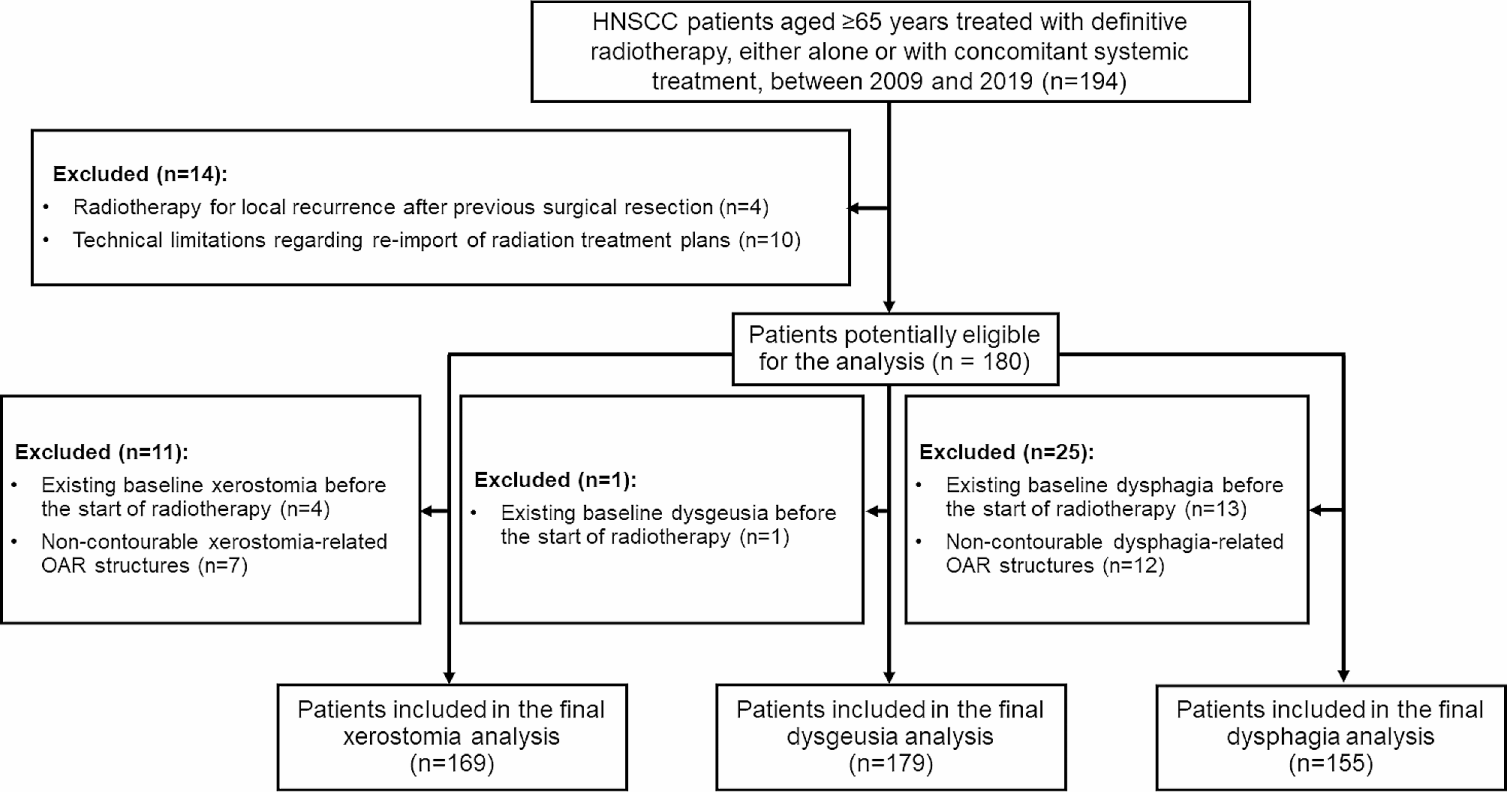
CONSORT diagram showing the inclusion and exclusion criteria of the analysis. HNSCC, head and neck squamous cell carcinoma

The study population comprised patients treated at the Department of Radiation Oncology, University Medical Center Freiburg, Germany, between July 2009 and November 2019. Patients were eligible for this retrospective analysis if they met the following criteria: (i) diagnosis of squamous cell carcinoma originating in the head and neck region, (ii) age of ≥ 65 years at the time of radiotherapy, and (iii) treatment with definitive radiotherapy (Fig. 1). The Ethics Committee of the University of Freiburg Medical Center approved this study (551/18).

Patient and treatment data were collected in a clinical documentation tool (MOSAIQ Oncology Management System, Elekta, Sunnyvale, CA, USA). This documentation covered patient and treatment characteristics, as well as reported radiotherapy-induced toxicities during and after radiotherapy. All patients underwent regular follow-up imaging and clinical evaluations, including evaluation of toxicities in three-monthly intervals during the first two years, six-monthly intervals in the third year, and annually in the fourth and fifth year after radiotherapy.

Patients underwent a planning CT scan in treatment position including an individually molded thermoplastic mask. If there were no contraindications, intravenous iodinated contrast media was administered immediately prior to the radiotherapy planning CT. Target volume definition and plan review was carried out by at least two board-certified radiation oncologists. If available, magnetic resonance imaging and positron emission tomography scans were co-registered with the planning CT and used for gross target volume (GTV) delineation of the primary tumor and metastatic lymph nodes. The high-risk clinical target volume (CTV) comprised the primary and nodal GTVs, added with a 5–8 mm margin but cropped by anatomic barriers. In some cases, an intermediate-risk CTV was contoured consisting of small but suspicious lymph nodes with a 5 mm margin. Elective nodal areas were treated, as per the international consensus guidelines [28–30]. A 5 mm margin was subsequently added for the planning target volumes (PTVs).

All patients in this analysis received definitive radiotherapy, either conventional three-dimensional conformal radiotherapy in the first few years of the analyzed time span, or IMRT, volumetric modulated arc therapy (VMAT) and helical tomotherapy later. If there were no contraindications, all patients with locoregionally advanced HNSCC received concomitant cisplatin. In case of contraindications against cisplatin, either cetuximab or other chemotherapy regimens such as carboplatin were used. Radiotherapy alone was used in patients with poor performance status or in patients who refused concomitant chemotherapy. Oncentra® External Beam (Nucletron B.V., The Netherlands) or Eclipse (Varian Medical Systems Inc., USA) were used for radiation treatment planning. While a sequential boost concept was conducted until 2018, a simultaneous integrated boost technique was introduced and integrated into clinical practice since then. Low-risk PTV usually received a dose of 50–54 Gy, while intermediate-risk PTV was treated with about 60 Gy, and high-risk PTV with about 66–70 Gy.

### Delineation of organs at risk

**Fig. 2:**
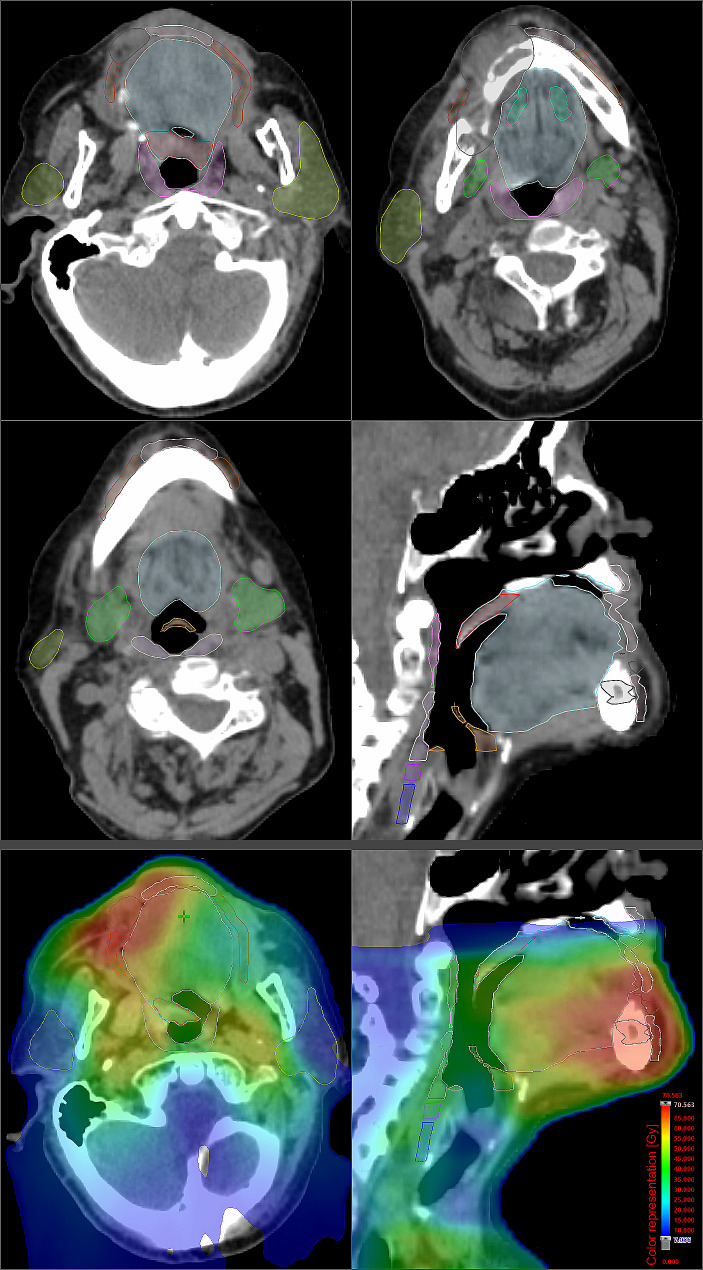
Organs at risks analyzed regarding chronic xerostomia, dysgeusia and dysphagia in older adults with head and neck squamous cell carcinoma undergoing definitive radiotherapy. Top: Axial and sagittal views of a radiation treatment planning CT, showing the contoured organs at risk: left and right parotid glands (chartreuse), left and right submandibular glands (green), left sublingual gland (turquoise), salivary glands of the left and right buccal mucosa (brown), salivary glands of the labial mucosa (pale pink), soft palate (red), extended oral cavity (light blue), supraglottic larynx (orange), superior pharyngeal constrictors (pink), middle pharyngeal constrictors (lilac), inferior pharyngeal constrictors (violet), cricopharyngeal muscles (dark violet). The gross tumor volume is shown in dark blue. Bottom: Color representation of the dose distribution in axial and sagittal sections of the aforementioned radiation treatment planning CT.

To improve the consistency of delineation accuracy for the analyzed OARs in this study, contouring of all analyzed OARs was again carried out based on a combination of recently published guidelines (Fig. 2) [31–33]. OARs commonly associated with xerostomia, including the parotid, submandibular, and sublingual glands, along with the minor salivary glands located in the soft palate, inner surface of the lips, and left and right buccal mucosa, were delineated following the guidelines described by van de Water et al. [31]. The swallowing-related OARs encompassed the superior, middle, and inferior PCMs, the cricopharyngeal muscle and the supraglottic larynx, delineated according to the guidelines by Christianen et al. [32]. The extended oral cavity was delineated in accordance with international consensus guidelines published by Brouwer and colleagues [33]. In cases where images were co-registered, OAR delineation was performed separately for each image series to account for morphological changes in the aforementioned organs or discrepancies in patient positioning between images.

### Endpoints

Three distinct endpoints were analyzed in this study: (1) chronic grade 2 xerostomia, (2) chronic grade 2 dysgeusia, and (3) chronic grade 2 or 3 dysphagia, according to the Common Terminology Criteria for Adverse Events (CTCAE) v5.0. Percutaneous endoscopic gastrostomy dependence was classified as dysphagia grade 3. Toxicities were considered as chronic if present at ≥ 90 days after completing radiotherapy, and the worst chronic toxicity grade documented during the follow-up period was used for the analyses. Evaluation of toxicity grading was performed by radiation oncologists, taking into account medical records, physical examination findings and patient-reported symptoms.

In order to only focus on radiotherapy-induced toxicities, patients with already present xerostomia, dysgeusia or dysphagia at baseline were excluded from the analysis. Additionally, patients with previous treatments to OARs, e.g., salivary gland resection, making delineation and dose calculation of the analyzed OARs not possible, were also excluded from the analysis.

### NTCP analyses

Dose matrices were converted to equivalent dose at 2 Gy fractions (EQD2) using an α/β value of 3 Gy for late effects, as also performed in previous analyses for chronic toxicities [34]. The summation of treatment plans was performed based on the transformed EQD2 dose matrices. Volumetric and dosimetric indices were extracted from Eclipse VARIAN TPS v.15.6 using a custom C#.NET application. The application was developed in-house and relied on ESAPICommander (https://github.com/isachpaz/ESAPICommander) which utilizes the VARIAN Eclipse Scripting Application Interface (ESAPI - https://varianapis.github.io/) [35]. Throughout this study, any mention of the term ‘*dose*’ exclusively pertains to the equivalent dose at 2 Gy fractions.

Multivariate imputation by chained equations was performed 10 times regarding the missing outcome variables for the analyzed toxicities as recommended by van den Bosch et al. and by using the R-package *mice* [36, 37]. To address the issue of multicollinearity between predictor variables, a Pearson correlation matrix that included all dose volume histogram parameters for the OARs was created. High correlations were found between the D_min_, D_max_, D_median_, D_98%_, D_2%_ and the D_mean_ within almost all OARs. As a result, only the mean doses were selected to enter the analyses. Univariate regression analyses were conducted to determine which representation of any paired or sequential organ should be included in the model-building process. To develop prediction models for each of the three endpoints, multivariable logistic regression analyses with Bayesian Information Criterion (BIC) forward stepwise variable selection were performed using R version 4.3.0 with the publicly available protocol and R codes [36]. In general, we followed the previously published recommendations from van den Bosch et al. [36]. The predicted NTCP for each toxicity endpoint was calculated based on the logistic regression model using the formula [38]:

$$ NTCP=P\left(Y\right)={(1+{e}^{-S})}^{-1}$$

where

$$ S={\beta }_{0}+\sum _{i=1}^{n}{\beta }_{i}\bullet {x}_{i}$$

and β_i_ are the regression coefficients and x_i_ are the distinct independent variables.

Internal validation of the developed NTCP models was subsequently performed using the bootstrapping method with 1000 iterations. Discrimination was quantified with the area under the curve (AUC) values, and calibration with the Hosmer–Lemeshow test, the corrected intercept of the calibration curve, and the corrected slope of the calibration curve.

## Results

The analyzed study population consisted of 180 patients with a median age of 73 years (Table 1). Tumors of the oropharynx and larynx were the most common (*n* = 69 (38%) and *n* = 37 (21%), respectively). The majority received concomitant systemic treatment (*n* = 126 (70%) with chemotherapy, *n* = 2 (1%) with cetuximab). A total of 73 patients developed chronic moderate xerostomia (grade 2), 34 moderate dysgeusia (grade 2), and 59 moderate-to-severe (grade 2–3) dysphagia (Table 2).

**Table 1:** Patient and tumor characteristics of the analyzed cohort (*n* = 158). Patients were treated with definitive radiotherapy between 2009 and 2019. TNM and UICC classification is based on the 7th UICC/AJCC TNM Staging System. ECOG, Eastern Cooperative Oncology Group; HPV, human papillomavirus; UICC, Union for International Cancer Control

Age [years]		Median (min-max)	
		73 (65–92)	
		n	%
Gender	Male	130	72
	Female	50	28
Smoking status	Non-smoker	48	27
	Smoker	108	60
	Unknown	24	13
ECOG	ECOG 0	93	52
	ECOG 1	67	37
	ECOG 2	20	11
Localization	Nasopharynx	4	2
	Oropharynx	69	38
	Hypopharynx	30	17
	Oral cavity	21	12
	Larynx	37	21
	Multilevel	12	7
	Salivary glands	3	2
	Other	4	2
UICC	I	13	7
	II	10	6
	III	24	13
	IV	133	74
cT	cT1	15	8
	cT2	25	14
	cT3	63	35
	cT4	77	43
cN	cN0	53	29
	cN1	10	6
	cN2a	11	6
	cN2b	52	29
	cN2c	44	24
	cN3	10	6
cM	cM0	160	89
	cM1	11	6
	cMx	9	5
HPV-status	HPV-negative	48	27
	HPV-positive	30	17
	Unknown	102	57
Radiotherapy completed	Not completed	25	14
	Completed	155	86
Concomitant systemic treatment	No chemotherapy	52	29
	Chemotherapy	126	70
	Cetuximab	2	1
Prescribed total dose [Gy]		**Median (min-max)**	
		70.0 (49.8–79.2)	

**Table 2:** Number of patients suffering from chronic xerostomia, dysgeusia and dysphagia. CTCAE, Common Terminology Criteria for Adverse Events, N/A, not available (due to death within the first 90 days after radiotherapy, insufficient documentation in the follow-up appointments, or refusal of the follow-up consultation).

CTCAE grade	Xerostomia	Dysgeusia	Dysphagia
0	35	59	51
1	35	52	24
2	73	34	40
3	0	0	19
4	0	0	0
5	0	0	0
N/A	28	34	28

In the univariate regression analyses, the EQD2 doses to the soft palate (OR = 1.028), the submandibular glands (ipsilateral: OR = 1.023, contralateral: OR = 1.024, combined: OR = 1.013), the contralateral parotid gland (OR = 1.068), the combined parotid glands (OR = 1.021), the contralateral sublingual gland (OR = 1.018), the combined sublingual gland (OR = 1.009), the salivary glands of the buccal mucosa (ipsilateral: OR = 1.023, contralateral: OR = 1.025, combined: OR = 1.012), and the salivary glands of the labial mucosa (OR = 1.037) were all significantly associated with moderate xerostomia (supplementary Table 1). Both the soft palate dose (OR = 1.023) and the extended oral cavity dose (OR = 1.027) were associated with chronic moderate dysgeusia. However, given the very high correlation (*r* >.85) between these two OARs, only the soft palate was included in the multivariable regression analysis, as model performance was superior with this approach. The EQD2 doses to the soft palate (OR = 1.027), extended oral cavity (OR = 1.032), superior PCM (OR = 1.029), middle PCM (OR = 1.029), and combined PCM (OR = 1.033) were related to moderate-to-severe dysphagia in the univariable regression. In the multivariate analysis, the EQD2 dose administered to the soft palate remained the only significant variable for all analyzed toxicities (xerostomia: OR = 1.028, dysgeusia: OR = 1.022, dysphagia: OR = 1.027). In terms of chronic moderate-to-severe dysphagia, the superior PCM remained as further independent variable (OR = 1.030), while all other variables were not significantly associated with the analyzed toxicities in the multivariable regression analyses.

Median value of D50% (average EQD2 dose) for the soft palate were 54.0 Gy in patients suffering from moderate xerostomia, 56.6 Gy in patients with moderate dysgeusia, and 54.6 Gy in patients with moderate-to-severe dysphagia, whereas patients without these toxicities had soft palate doses of 37.5 Gy (xerostomia grade 0–1), 46.2 Gy (dysgeusia grade 0–1), and 37.2 Gy (dysphagia grade 0–1), respectively (Table 3). The superior PCM was exposed to a median of 61.4 Gy (D50% EQD2 dose) in patients with chronic grade 2–3 dysphagia, and only 53.6 Gy in patients with chronic grade 0–1 dysphagia.

**Table 3:** Median value of D50% (average EQD2 dose with α/ß = 3 Gy) of the analyzed organ at risks depending on the development of treatment-related toxicities. CTCAE, Common Terminology Criteria of Adverse Events; PCM, pharyngeal constrictor muscle

Organ at risk	Median average (mean) EQD2 dose (α/ß = 3 Gy) [Gy]					
	Xerostomia CTCAE grade 2		Dysgeusia CTCAE grade 2		Dysphagia CTCAE grade 2/3	
	Yes	No	Yes	No	Yes	No
Ipsilateral parotid gland	23.0	17.8	20.5	20.6		
Contralateral parotid gland	17.6	15.2	17.2	16.2		
Ipsilateral submandibular gland	65.3	63.9	63.7	64.8		
Contralateral submandibular gland	59.6	53.5	56.2	56.5		
Ipsilateral sublingual gland	43.4	42.3				
Contralateral sublingual gland	38.9	37.0				
Salivary glands of the ipsilateral buccal mucosa	31.4	27.1				
Salivary glands of the contralateral buccal mucosa	30.1	23.4				
Salivary glands of the labial mucosa	18.7	14.9				
Soft palate	54.0	37.5	56.6	46.2	54.6	37.2
Extended oral cavity			50.5	44.0		
Superior PCM					61.4	53.6
Middle PCM					64.7	61.1
Inferior PCM					53.7	57.2
Supraglottic larynx					63.8	60.0
Cricopharyngeal muscle					45.2	46.5

**Fig. 3:**
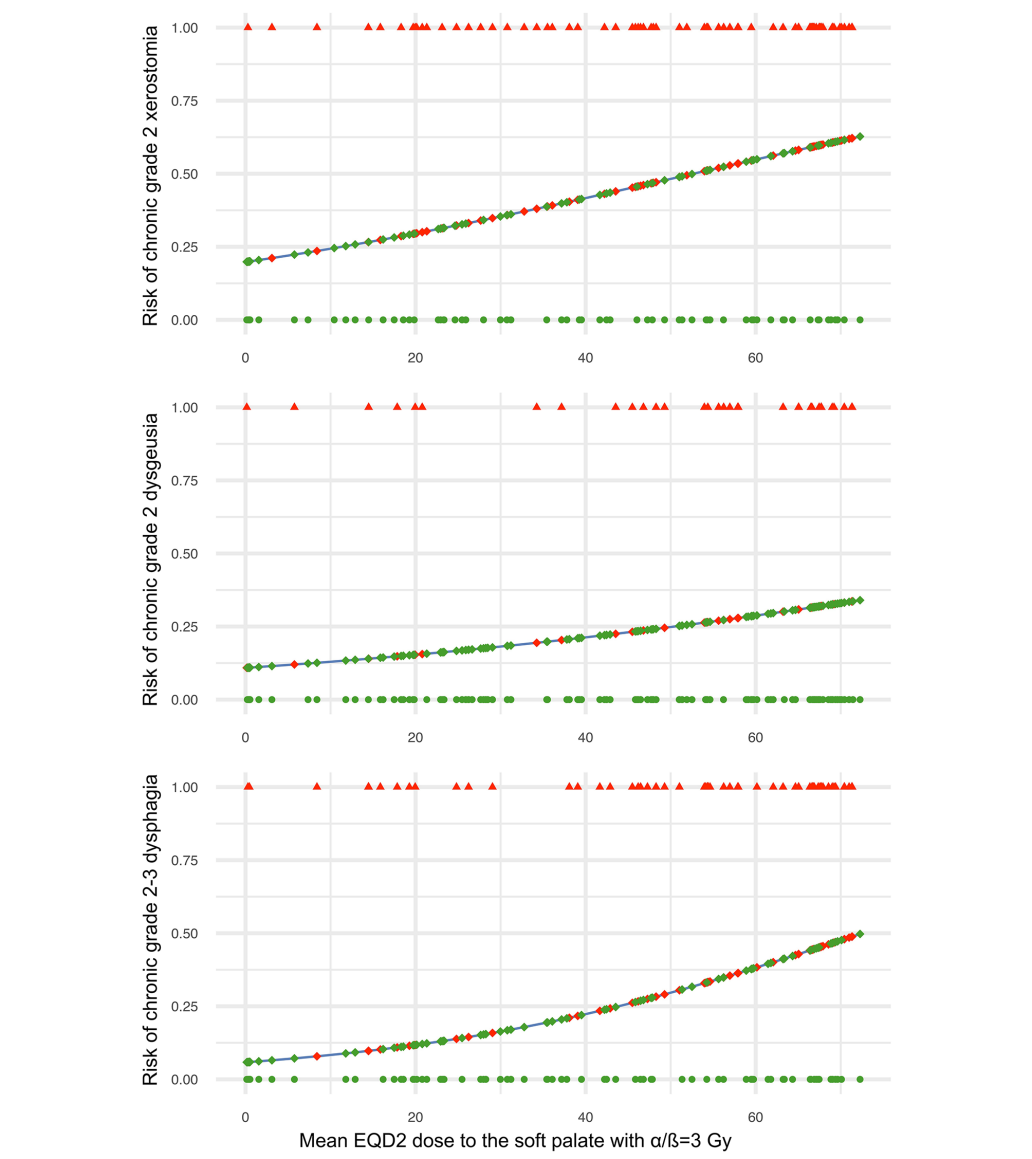
Normal tissue complication probability models for chronic moderate xerostomia, moderate dysgeusia and moderate-to-severe dysphagia based on the soft palate dose. NTCP, normal tissue complication probability; NTCP models for xerostomia (top), dysgeusia (middle) and dysphagia (bottom) are shown. The formula *NTCP*= *P(Y)*=(1+*e*^-*S*^)^-1^ with $S={\beta }_{0}+\sum _{i=1}^{n}{\beta }_{i}\bullet {x}_{i}$ was used for the NTCP analyses. The blue line represents the NTCP curve, calculated for each patient using the logistic regression analysis formula. Green dots indicate the mean soft palate EQD2 (α/ß=3 Gy) dose for individual patients who did not exhibit any of the investigated toxicities projected onto the NTCP curve as green diamonds. Red triangles represent the mean soft palate EQD2 (α/ß=3 Gy) dose of patients with documented toxicity, projected onto the NTCP curve as red diamonds.

The resulting NTCP models are shown in Fig. 3. The soft palate dose resulting in a 50% risk for moderate xerostomia and moderate-to-severe dysphagia was 44.6 and 57.5 Gy, respectively, while the soft palate dose associated with a 25% risk of moderate dysgeusia was 50.6 Gy. Indicators regarding the performance of the NTCP models are shown in Table 4. The optimism-corrected AUC values of the NTCP model based on the bootstrapping technique [36] were 0.64 for xerostomia, 0.60 for dysgeusia, and 0.64 for dysphagia. The Hosmer-Lemeshow tests showed a significant agreement between predicted risk and observed toxicity outcome for the NTCP models (*p* >.05). Given the AUC values derived from the development cohort (xerostomia: AUC = 0.66, dysgeusia: AUC = 0.63, dysphagia: AUC = 0.66), the estimated optimism values of the AUC were 0.02 for xerostomia, 0.03 for dysgeusia, and 0.02 for dysphagia.

**Table 4:** Model performance and calibration for the normal tissue complication probability models for chronic moderate xerostomia (grade 2), moderate dysgeusia (grade 2) and moderate-to-severe dysphagia (grade 2 or 3). AUC, area under the curve

Performance measure		Xerostomia	Dysgeusia	Dysphagia
Discrimination	Mean AUC	0.66	0.63	0.66
	Corrected AUC	0.64	0.60	0.64
Calibration	Hosmer–Lemeshow test	*X*^*2*^ = 6.549 (*p* =.586)	*X*^*2*^ = 12.931 (*p* =.114)	*X*^*2*^ = 4.980 (*p* =.760)
	Corrected intercept of calibration curve	0.007	0.005	−0.203
	Corrected slope of calibration curve	0.870	0.989	0.826

## Discussion

In this analysis of a large tertiary cancer center, the soft palate dose was associated with moderate xerostomia, moderate dysgeusia, and moderate-to-severe dysphagia in older HNSCC patients receiving definitive radiotherapy. The developed and internally validated NTCP models exhibited a moderate performance in predicting these chronic toxicities.

The fact that the soft palate dose was associated with all three analyzed toxicities, even though the pathophysiology of these toxicities is different, is worth mentioning. As minor salivary glands are located at the soft palate, this may explain the soft palate’s attribution to the development of xerostomia. While the major salivary glands produce the majority of saliva during eating, the minor salivary glands predominantly produce saliva during sleep, which is why minor salivary gland dysfunction potentially affects patient-reported xerostomia at night [39]. Taste variations are in part also attributed to reduced saliva production, so that the association between the soft palate dose and dysgeusia may also be related to this fact. In line with our findings, the soft palate was associated with sticky saliva at six months after radiotherapy in the NTCP model reported by Beetz and colleagues [24]. In addition, the soft palate itself has been shown to exhibit a gustatory function which is independent of the tongue [40]. In terms of dysphagia, the soft palate plays a crucial role in the oral preparatory and oropharyngeal phase of swallowing [41] and therefore has been investigated in other analyses regarding potential dysphagia-related OARs [42].

In the CITOR profile based on a longitudinal risk prediction analysis of 22 common radiotherapy-induced toxicities, the oral cavity was the predominant OAR that was associated with 12 toxicities [26]. Similarly, the mean dose to the oral cavity was related to chronic xerostomia and dysgeusia after chemoradiation in a de-escalation study for human papillomavirus (HPV)-positive oropharyngeal cancer patients [43], and we also observed a significant correlation between the oral cavity dose and chronic dysgeusia/dysphagia in the univariate regression analyses. However, we had a high correlation between the extended oral cavity and the soft palate, and the model performance was slightly superior with the soft palate instead the extended oral cavity OAR, so that only the soft palate was entered into the multivariable regression model. It should be noted in this context that the extended oral cavity, when contoured according to the guideline by Brouwers et al. [33], contains the soft palate.

We also found a significant association between the superior PCM dose and dysphagia in the multivariable regression. This is in line with several analyses, e.g., analyses from Mazzola et al. [44], Levendag et al. [45], and Mortensen et al. [46] who observed a significant association between the superior PCM dose and dysphagia. There is randomized phase III evidence that dysphagia-optimized IMRT, i.e., reducing the dose to the PCMs, significantly improves swallowing function in patients undergoing radiotherapy [12]. It should be noted that patients analyzed in our study were exposed to considerably higher PCM doses than required in the PCM-sparing protocols, e.g., < 50 Gy (physical dose) of the PCM excluding the overlapping part with the high-dose CTV in the DARS study [47]. The DAHANCA Radiotherapy Guideline 2020 guideline indicates a dose constraint of D_mean_ <55 Gy for the superior, middle, and inferior PCM [48].

Contrary to the parotid glands and PCMs, the soft palate is not routinely spared in the radiation treatment planning process, and efforts in reducing the parotid gland dose by using highly modulated radiation techniques may result in higher doses to the soft palate [25]. For instance, the randomized COSTAR phase III trial in which the value of cochlea-sparing IMRT was tested in parotid cancer patients reported a higher incidence of late xerostomia in the cochlea-sparing IMRT than in the three-dimensional conformal radiotherapy group, probably due to higher low-dose volumes in the oral cavity and oropharynx, thereby affecting the small salivary glands [49]. There are several strategies to reduce the soft palate dose, e.g., reducing CTV-PTV margins accompanied by daily image guidance [50, 51], omitting treatment of cervical lymph node level VII in cases in which it is possible according to current consensus recommendations [52, 53], or omitting contralateral neck irradiation in well-lateralized oropharyngeal cancers [54, 55].

Although presenting one of the first NTCP analyses focusing on older HNSCC patients, our analysis has some limitations. We only used physician-assessed but not patient-reported toxicities as endpoints, as patient-reported outcomes were only available for a minority of patients. In the future, patient self-reported outcomes should be also collected, as physicians are known to underestimate the severity of patient-reported toxicities [56]. Furthermore, we included patients treated between 2009 and 2019, resulting in heterogeneity regarding the applied radiotherapy treatment techniques. In order to ensure sufficient statistical power for the analyses, we however decided to include all patients from this time span.

## Conclusions

The soft palate was found to be an important OAR in terms of chronic xerostomia, dysgeusia and dysphagia in older adults with HNSCC undergoing definitive radiotherapy. External validation on the basis of patient-reported outcomes is warranted to verify our findings.

### Electronic supplementary material

Below is the link to the electronic supplementary material.

**Univariable regression analyses**

## Data Availability

The data that support the findings of this study are available from the corresponding author upon reasonable request.

## References

[1] Sung H, Ferlay J, Siegel RL, Laversanne M, Soerjomataram I, Jemal A et al. Global Cancer Statistics 2020: GLOBOCAN Estimates of Incidence and Mortality Worldwide for 36 Cancers in 185 Countries. CA: A Cancer Journal for Clinicians. 2021;71:209– 49. 10.3322/caac.21660 33538338

[2] Dickstein DR, Lehrer EJ, Hsieh K, Hotca A, Jones BM, Powers A et al. Management of older adults with locally Advanced Head and Neck Cancer. Cancers (Basel). 2022;14. 10.3390/cancers14112809 PMC9179912 35681789

[3] Gormley M, Creaney G, Schache A, Ingarfield K, Conway DI (2022). Reviewing the epidemiology of head and neck cancer: definitions, trends and risk factors. Br Dent J.

[4] Gatta G, Capocaccia R, Botta L. Descriptive epidemiology of the head and neck cancers in old patients. Front Oncol. 2023;13. 10.3389/fonc.2023.1102236 PMC10247222 37293589

[5] National Comprehensive Cancer Network. Head and Neck Cancer (Version 2.2023). 2023.

[6] Lacas B, Carmel A, Landais C, Wong SJ, Licitra L, Tobias JS et al. Meta-analysis of chemotherapy in head and neck cancer (MACH-NC): an update on 107 randomized trials and 19805 patients, on behalf of MACH-NC group. Radiother Oncol. 2021. 10.1016/j.radonc.2021.01.013 PMC8386522 33515668

[7] Rühle A, Marschner S, Haderlein M, Fabian A, Weymann M, Behrens M (2023). Evaluation of concomitant systemic treatment in older adults with Head and Neck squamous cell carcinoma undergoing definitive Radiotherapy. JAMA Netw Open.

[8] Amini A, Jones BL, McDermott JD, Serracino HS, Jimeno A, Raben D (2016). Survival outcomes with concurrent chemoradiation for elderly patients with locally advanced head and neck cancer according to the National Cancer Data Base. Cancer.

[9] Rühle A, Haehl E, Kalckreuth T, Stoian R, Spohn SKB, Sprave T (2021). Surviving Elderly patients with Head-and-Neck squamous cell carcinoma—what is the long-term quality of life after curative Radiotherapy?. Cancers.

[10] McDowell L, Rischin D, Gough K, Henson C (2022). Health-Related Quality of Life, Psychosocial Distress and Unmet needs in older patients with Head and Neck Cancer. Front Oncol.

[11] Nutting CM, Morden JP, Harrington KJ, Urbano TG, Bhide SA, Clark C (2011). Parotid-sparing intensity modulated versus conventional radiotherapy in head and neck cancer (PARSPORT): a phase 3 multicentre randomised controlled trial. Lancet Oncol.

[12] Nutting C, Finneran L, Roe J, Sydenham MA, Beasley M, Bhide S et al. Dysphagia-optimised intensity-modulated radiotherapy versus standard intensity-modulated radiotherapy in patients with head and neck cancer (DARS): a phase 3, multicentre, randomised, controlled trial. Lancet Oncol. 2023. 10.1016/S1470-2045(23)00265-6 37423227

[13] Li X, Kitpanit S, Lee A, Mah D, Sine K, Sherman EJ (2021). Toxicity profiles and survival outcomes among patients with nonmetastatic nasopharyngeal carcinoma treated with intensity-modulated Proton Therapy vs intensity-modulated Radiation Therapy. JAMA Netw Open.

[14] van der Laan HP, van de Water TA, van Herpt HE, Christianen ME, Bijl HP, Korevaar EW (2013). The potential of intensity-modulated proton radiotherapy to reduce swallowing dysfunction in the treatment of head and neck cancer: a planning comparative study. Acta Oncol.

[15] Taylor KJ, Amdal CD, Bjordal K, Astrup GL, Herlofson BB, Duprez F et al. Serious Long-Term Effects of Head and Neck Cancer from the Survivors’ Point of View. Healthc (Basel). 2023;11. 10.3390/healthcare11060906 PMC10048748 36981562

[16] Irune E, Dwivedi RC, Nutting CM, Harrington KJ (2014). Treatment-related dysgeusia in head and neck cancer patients. Cancer Treat Rev.

[17] Stieb S, Mohamed ASR, Deshpande TS, Harp J, Greiner B, Garden AS (2020). Prospective observational evaluation of radiation-induced late taste impairment kinetics in oropharyngeal cancer patients: potential for improvement over time?. Clin Translational Radiation Oncol.

[18] Machtay M, Moughan J, Trotti A, Garden AS, Weber RS, Cooper JS (2008). Factors associated with severe late toxicity after concurrent chemoradiation for locally advanced head and neck cancer: an RTOG analysis. J Clin Oncol.

[19] Johansson A-K, Omar R, Mastrovito B, Sannevik J, Carlsson GE, Johansson A (2022). Prediction of xerostomia in a 75-year-old population: a 25-year longitudinal study. J Dent.

[20] Doan TN, Ho WC, Wang LH, Chang FC, Nhu NT, Chou LW. Prevalence and Methods for Assessment of Oropharyngeal Dysphagia in older adults: a systematic review and Meta-analysis. J Clin Med. 2022;11. 10.3390/jcm11092605 PMC9104951 35566731

[21] Nakazato M, Endo S, Yoshimura I, Tomita H. Influence of aging on electrogustometry thresholds. Acta Otolaryngol Suppl. 2002:16–26. 10.1080/00016480260046382 12132616

[22] Vandenberghe-Descamps M, Labouré H, Prot A, Septier C, Tournier C, Feron G (2016). Salivary Flow decreases in healthy Elderly people independently of Dental Status and Drug Intake. J Texture Stud.

[23] Sommers L, van der Laan HP, Van den Bosch L, Hoek J, Zon-Meijer T, Oldehinkel E (2022). OC-0436 External validation of NTCP models for dysphagia and xerostomia in the elderly patients with HNSCC. Radiother Oncol.

[24] Beetz I, Schilstra C, van der Schaaf A, van den Heuvel ER, Doornaert P, van Luijk P (2012). NTCP models for patient-rated xerostomia and sticky saliva after treatment with intensity modulated radiotherapy for head and neck cancer: the role of dosimetric and clinical factors. Radiother Oncol.

[25] Beetz I, Schilstra C, van Luijk P, Christianen MEMC, Doornaert P, Bijl HP (2012). External validation of three dimensional conformal radiotherapy based NTCP models for patient-rated xerostomia and sticky saliva among patients treated with intensity modulated radiotherapy. Radiother Oncol.

[26] Van den Bosch L, van der Schaaf A, van der Laan HP, Hoebers FJP, Wijers OB, van den Hoek JGM (2021). Comprehensive toxicity risk profiling in radiation therapy for head and neck cancer: a new concept for individually optimised treatment. Radiother Oncol.

[27] Samant P, Ruysscher Dd, Hoebers F, Canters R, Hall E, Nutting C et al. Machine learning for normal tissue complication probability prediction: predictive power with versatility and easy implementation. Clin Translational Radiation Oncol. 2023;39. 10.1016/j.ctro.2023.100595 PMC9984444 36880063

[28] Grégoire V, Ang K, Budach W, Grau C, Hamoir M, Langendijk JA (2014). Delineation of the neck node levels for head and neck tumors: a 2013 update. DAHANCA, EORTC, HKNPCSG, NCIC CTG, NCRI, RTOG, TROG consensus guidelines. Radiother Oncol.

[29] Grégoire V, Daisne JF, Geets X, Levendag P (2003). Selection and delineation of target volumes in head and neck tumors: beyond ICRU definition. Rays.

[30] Grégoire V, Evans M, Le QT, Bourhis J, Budach V, Chen A (2018). Delineation of the primary tumour clinical target volumes (CTV-P) in laryngeal, hypopharyngeal, oropharyngeal and oral cavity squamous cell carcinoma: AIRO, CACA, DAHANCA, EORTC, GEORCC, GORTEC, HKNPCSG, HNCIG, IAG-KHT, LPRHHT, NCIC CTG, NCRI, NRG Oncology, PHNS, SBRT, SOMERA, SRO, SSHNO, TROG consensus guidelines. Radiother Oncol.

[31] van de Water TA, Bijl HP, Westerlaan HE, Langendijk JA (2009). Delineation guidelines for organs at risk involved in radiation-induced salivary dysfunction and xerostomia. Radiother Oncol.

[32] Christianen ME, Langendijk JA, Westerlaan HE, van de Water TA, Bijl HP (2011). Delineation of organs at risk involved in swallowing for radiotherapy treatment planning. Radiother Oncol.

[33] Brouwer CL, Steenbakkers RJHM, Bourhis J, Budach W, Grau C, Grégoire V (2015). CT-based delineation of organs at risk in the head and neck region: DAHANCA, EORTC, GORTEC, HKNPCSG, NCIC CTG, NCRI, NRG Oncology and TROG consensus guidelines. Radiother Oncol.

[34] Vasquez Osorio E, Abravan A, Green A, van Herk M, Lee LW, Ganderton D et al. Dysphagia at 1 year is Associated with Mean dose to the Inferior section of the brain stem. Int J Radiat Oncol Biol Phys. 2023. 10.1016/j.ijrobp.2023.06.004 PMC10581448 37331569

[35] Hojin K, Jungwon K, Chiyoung J, Byungchul C (2017). Institutional applications of Eclipse Scripting Programming Interface to Clinical workflows in Radiation Oncology. Prog Med Phys.

[36] Van den Bosch L, Schuit E, van der Laan HP, Reitsma JB, Moons KGM, Steenbakkers RJHM (2020). Key challenges in normal tissue complication probability model development and validation: towards a comprehensive strategy. Radiother Oncol.

[37] van Buuren S, Groothuis-Oudshoorn K (2011). Mice: multivariate imputation by chained equations in R. J Stat Softw.

[38] Stieb S, Lee A, van Dijk LV, Frank S, Fuller CD, Blanchard P (2021). NTCP modeling of Late effects for Head and Neck Cancer: a systematic review. Int J Part Ther.

[39] Ferguson DB, Botchway CA (1979). Circadian variations in flow rate and composition of human stimulated submandibular saliva. Arch Oral Biol.

[40] Ikeda M, Ikui A, Tomita H. Gustatory function of the soft palate. Acta Otolaryngol Suppl. 2002:69–73. 10.1080/00016480260046436 12132623

[41] Russi EG, Corvò R, Merlotti A, Alterio D, Franco P, Pergolizzi S (2012). Swallowing dysfunction in head and neck cancer patients treated by radiotherapy: review and recommendations of the supportive task group of the Italian Association of Radiation Oncology. Cancer Treat Rev.

[42] Hedström J, Tuomi L, Finizia C, Olsson C (2019). Identifying organs at risk for radiation-induced late dysphagia in head and neck cancer patients. Clin Translational Radiation Oncol.

[43] Fried DV, Das SK, Shen C, Marks LB, Chera BS (2022). Impact of oral cavity dosimetry on patient reported Xerostomia and Dysgeusia in the setting of Deintensified Chemoradiotherapy. Adv Radiat Oncol.

[44] Mazzola R, Ricchetti F, Fiorentino A, Fersino S, Giaj Levra N, Naccarato S (2014). Dose-volume-related dysphagia after constrictor muscles definition in head and neck cancer intensity-modulated radiation treatment. Br J Radiol.

[45] Levendag PC, Teguh DN, Voet P, van der Est H, Noever I, de Kruijf WJ (2007). Dysphagia disorders in patients with cancer of the oropharynx are significantly affected by the radiation therapy dose to the superior and middle constrictor muscle: a dose-effect relationship. Radiother Oncol.

[46] Mortensen HR, Jensen K, Aksglæde K, Behrens M, Grau C (2013). Late dysphagia after IMRT for head and neck cancer and correlation with dose-volume parameters. Radiother Oncol.

[47] Petkar I, Rooney K, Roe JWG, Patterson JM, Bernstein D, Tyler JM (2016). DARS: a phase III randomised multicentre study of dysphagia- optimised intensity- modulated radiotherapy (Do-IMRT) versus standard intensity- modulated radiotherapy (S-IMRT) in head and neck cancer. BMC Cancer.

[48] Jensen K, Friborg J, Hansen CR, Samsøe E, Johansen J, Andersen M (2020). The Danish Head and Neck Cancer Group (DAHANCA) 2020 radiotherapy guidelines. Radiother Oncol.

[49] Nutting CM, Morden JP, Beasley M, Bhide S, Cook A, De Winton E (2018). Results of a multicentre randomised controlled trial of cochlear-sparing intensity-modulated radiotherapy versus conventional radiotherapy in patients with parotid cancer (COSTAR; CRUK/08/004). Eur J Cancer.

[50] Navran A, Heemsbergen W, Janssen T, Hamming-Vrieze O, Jonker M, Zuur C (2019). The impact of margin reduction on outcome and toxicity in head and neck cancer patients treated with image-guided volumetric modulated arc therapy (VMAT). Radiother Oncol.

[51] Rühle A, Grosu A-L, Nicolay NH (2021). De-escalation strategies of (Chemo)Radiation for Head-and-Neck squamous cell Cancers—HPV and Beyond. Cancers.

[52] Grégoire V, Grau C, Lapeyre M, Maingon P (2018). Target volume selection and delineation (T and N) for primary radiation treatment of oral cavity, oropharyngeal, hypopharyngeal and laryngeal squamous cell carcinoma. Oral Oncol.

[53] Biau J, Lapeyre M, Troussier I, Budach W, Giralt J, Grau C (2019). Selection of lymph node target volumes for definitive head and neck radiation therapy: a 2019 Update. Radiother Oncol.

[54] Sher DJ, Adelstein DJ, Bajaj GK, Brizel DM, Cohen EEW, Halthore A (2017). Radiation therapy for oropharyngeal squamous cell carcinoma: executive summary of an ASTRO evidence-based clinical practice Guideline. Pract Radiat Oncol.

[55] Koyfman SA, Ismaila N, Crook D, D’Cruz A, Rodriguez CP, Sher DJ (2019). Management of the Neck in squamous cell carcinoma of the oral cavity and oropharynx: ASCO Clinical Practice Guideline. J Clin Oncol.

[56] Meirovitz A, Murdoch-Kinch CA, Schipper M, Pan C, Eisbruch A (2006). Grading xerostomia by physicians or by patients after intensity-modulated radiotherapy of head-and-neck cancer. Int J Radiation Oncology*Biology*Physics.

