# Supplementary material for: The role of the soft palate dose regarding normal tissue toxicities in older adults with head and neck cancer undergoing definitive radiotherapy
Source: Radiat Oncol. 2024 Apr 30;19:53. doi: 10.1186/s13014-024-02426-5 (PMC11061999; doi:10.1186/s13014-024-02426-5)
Supplement: Supplementary file 1 — Univariable regression analyses [file 13014_2024_2426_MOESM1_ESM.docx]

**Supplementary table 1: Univariable regression analyses.** CTCAE, Common Terminology Criteria of Adverse Events; PCM, pharyngeal constrictor muscle.

| **Variable** | **Endpoints at 90 days after completion of radiotherapy** | | | | | | | | |
| --- | --- | --- | --- | --- | --- | --- | --- | --- | --- |
|  | **Xerostomia CTCAE grade 2** | | | **Dysgeusia CTCAE grade 2** | | | **Dysphagia CTCAE grade 2/3** | | |
|  | **OR** | **(95% CI)** | **p-value** | **OR** | **(95% CI)** | **p-value** | **OR** | **(95% CI)** | **p-value** |
| **Ipsilateral parotid gland** | 1.018 | (0.996-1.040) | 0.146 |  |  |  |  |  |  |
| **Contralateral parotid gland** | 1.068 | (1.025-1.113) | **0.006** |  |  |  |  |  |  |
| **Combined parotid glands** | 1.021 | (1.005-1.038) | **0.026** |  |  |  |  |  |  |
| **Ipsilateral submandibular gland** | 1.023 | (1.006-1.041) | **0.032** |  |  |  |  |  |  |
| **Contralateral submandibular gland** | 1.024 | (1.007-1.042) | **0.023** |  |  |  |  |  |  |
| **Combined submandibular glands** | 1.013 | (1.004-1.022) | **0.025** |  |  |  |  |  |  |
| **Ipsilateral sublingual gland** | 1.016 | (1.001-1.031) | 0.058 |  |  |  |  |  |  |
| **Contralateral sublingual gland** | 1.018 | (1.003-1.034) | **0.044** |  |  |  |  |  |  |
| **Combined sublingual glands** | 1.009 | (1.001-1.016) | **0.049** |  |  |  |  |  |  |
| **Salivary glands of the ipsilateral buccal mucosa** | 1.023 | (1.005-1.041) | **0.039** |  |  |  |  |  |  |
| **Salivary glands of the contralateral buccal mucosa** | 1.025 | (1.006-1.045) | **0.032** |  |  |  |  |  |  |
| **Combined salivary glands of the buccal mucosa** | 1.012 | (1.003-1.022) | **0.033** |  |  |  |  |  |  |
| **Salivary glands of the labial mucosa** | 1.037 | (1.007-1.068) | **0.042** |  |  |  |  |  |  |
| **Combined salivary glands of the oral mucosa** | 1.010 | (1.003-1.017) | **0.028** |  |  |  |  |  |  |
| **Soft palate** | 1.028 | (1.013-1.043) | **0.001** | 1.023 | (1.005-1.041) | **0.033** | 1.027 | (1.011-1.043) | **0.001** |
| **Extended oral cavity** |  |  |  | 1.027 | (1.006-1.050) | **0.036** | 1.032 | (1.013-1.051) | **0.003** |
| **Superior PCM** |  |  |  |  |  |  | 1.029 | (1.010-1.049) | **0.008** |
| **Middle PCM** |  |  |  |  |  |  | 1.029 | (1.007-1.052) | **0.029** |
| **Inferior PCM** |  |  |  |  |  |  | 1.003 | (0.985-1.022) | 0.780 |
| **Combined PCM (sup. + mid. + inf.)** |  |  |  |  |  |  | 1.033 | (1.010-1.058) | **0.017** |
| **Supraglottic larynx** |  |  |  |  |  |  | 1.017 | (0.996-1.039) | 0.181 |
| **Cricopharyngeal muscle** |  |  |  |  |  |  | 1.000 | (0.983-1.017) | 0.990 |
